# Steer clear of honorary authorship

**DOI:** 10.1093/jhps/hnz064

**Published:** 2020-01-30

**Authors:** Richard (Ricky) Villar

**Affiliations:** Editor-in-Chief, Journal of Hip Preservation Surgery

The last few months have been interesting, as I have discovered a new worry. Editors do spend many moments worrying. My concern has been the recent increase in authors seeking to add further names to papers once their work has been accepted. There have also been requests to change the order of authors after publication has been agreed. This rarely happens on submission, but sufficient requests landed on my desk in only a few weeks to make me ask around. It appears we are not alone. Several of my editor colleagues in journals elsewhere in the world are experiencing a similar dilemma.

The rules are clear. When a paper is submitted, the corresponding author declares that all the authors are happy to feature and that they played sufficient part in the process to justify inclusion [1]. It seems illogical to request that authors should be added after acceptance, especially for a journal that undertakes blinded review. One author, two authors, three, or a trillion, a reviewer will have no idea how many names are on a paper, or who they are, while the work is being appraised. Papers stand or fall by their content. It is simply the way it is done.

Authorship does matter, as it implies accountability for any published work. The International Committee of Medical Journal Editors (ICMJE) has laid down guidelines that most appear to follow [2]. According to ICMJE, authorship is based on the following four criteria:

- Substantial contributions to the conception or design of the work or the acquisition, analysis or interpretation of data for the work; and
- Drafting the work or revising it critically for important intellectual content; and
- Final approval of the version to be published; and
- Agreement to be accountable for all aspects of the work in ensuring that questions related to the accuracy or integrity of any part of the work are appropriately investigated and resolved.

Any researcher who does not meet these criteria, but who was nevertheless to some extent involved, should be acknowledged.

Simple? I would have thought so, but it appears some have difficulty with this, as the number of submissions where there is a request to change authors’ names and order after acceptance is increasing. I fail to understand why.

Despite the clear guidelines from the ICMJE, it appears that researchers still do not always fully comprehend what is expected of them. As recently as 2016, Bozeman and Youtie [3] concluded that there was no consensus on what type of contribution sufficed for a co-authorship award. To reach this decision, they interviewed 60 academic science or engineering researchers in 14 disciplines and also employed data from 161 website posts by 93 study participants.

Perhaps that is why, 2 years earlier, in 2014, Kennedy, Barnsteiner and Daly [4] reported that in the nursing literature the prevalence of honorary authorship was 42% and that of ghost authorship was 27.6%. These findings supported an even earlier (2011) report by Wislar *et al*. [5] in the British Medical Journal, to say there was evidence of honorary or ghost authorship in 21% of articles published in major medical journals. In the same year, similar findings were identified in three pharmacy journals by Dotson and Slaughter [6], who found honorary authorship in 14.3% of articles and ghost authorship in 0.9%. Honorary authorship was more common in original research than in review articles. Of interest, articles with honorary authors had longer by-lines than articles without honorary authors. Put simply, the more authors on a paper, the more likely it is that one or some will be honorary. The problem has manifestly existed for a considerable period. For example, Flanagin *et al*. [7], in 1998, established that in three peer-reviewed, large-circulation general medical journals, 19% of articles had evidence of honorary authorship, 11% had evidence of ghost authorship and 2% had evidence of both.

For clarity, a ghost author is one who might have made a significant contribution to a manuscript but there is no acknowledgement of their contribution [8]. An honorary author [9] is the same as a guest or gift author and is simply an author who has been added to a paper despite taking little or no part in the research leading to the paper being accepted.

Faced with this perplexing situation, the Committee on Publication Ethics (COPE) has produced guidelines and described example cases, so that authors and publishers have direction [10]. There are four authorship changes, excluding name reordering, that a journal might expect to receive:

- corresponding author requests addition of extra author before publication [11];
- corresponding author requests removal of author before publication[12];
- request for addition of extra author after publication [13];
- request for removal of author after publication [14].

The bottom-line decision for each of these scenarios is that all authors must agree before any authorship changes are made. For example, if a corresponding author submits a paper with five authors at the start, and then decides after acceptance that a further 20 names should be added—such things do happen—then the initial five authors must each agree that every one of the further 20 names can be added. Meanwhile the further 20 names must also be happy to be added as authors, and happy with everyone else featuring, too. Imagine the logistic workload required to seek these permissions. Truly, the simplest way to avoid this is to ensure that all authors are included from the start.

To an outsider, these systems may appear unnecessary but let us look at the issue in greater depth. Author manipulation, be it intentional or unintentional, can be a huge problem. Take the paper [15] that was retracted from an orthopaedic journal in 2017 as the submission’s data were used without the authority or permission of the co-authors [16]. Or, the manuscript that was submitted to a journal carrying the name of a well-known Dutch economist. The poor chap had no idea his name was being used and, when asked, added that it was the third time that year someone had submitted an article in his name without his knowledge [17]. Another publisher, again in The Netherlands, retracted 13 published studies with another 52 under consideration, after learning that someone illegally accessed its workflows to add fake authors and manipulate text [18]. A daughter of a high-profile South Korean official was quoted as a co-author [19] when she was still at high school, an American researcher once included his cat [20] (FDC Willard was the cat [21]), and another his dog [22] (Liboiron G, Grandmother Liboiron, was the dog [23]). Manifestly these are exceptional cases, but there is no doubt that authorship is also proliferating when compared with papers submitted some decades ago. Ojerholm and Swisher McClure, in 2015, looked at 2005 articles published in the field of radiation oncology between 1984 and 2014. They found that the mean number of authors per publication had more than doubled in 30 years [24]. Similar findings have also been seen in a major sports medicine journal by Schrock, Kraeutler and McCarty [25] who reported that in 1994 the mean number of authors per article was 3.8. By 2014 this had increased to 5.8, which they found to be statistically significant.

This may be more than about individual author status, as there may be impact-factor tactics at play as well, as exemplified by Huamaní *et al*. in 2015 [26]. They looked at scientific research in obstructive sleep apnea syndrome by undertaking an analysis of published work for the period 1991–2012. Looking at a total of 6896 articles, they found that the probability of citation increased by 1.23 times for each additional author and by 2.23 times for each additional country.

In summary, what harm can honorary or ghost authorship create? On the face of it, what is the problem? Think for a moment. Honorary authors carry accountability for the published work [27]. What would you do, as an honorary author, if the integrity of that work was called into question at some future date? What if you were the author who had undertaken the bulk of the work for a project? Would you really want all those extra names to dilute your own, hard efforts?

My advice is to approach any possible honorary authors before a project starts and ask them to play a part in the paper’s preparation. How difficult can that be? Should someone ask you to be an honorary author, think once, twice, as many times as you desire, but only accept authorship if you intend to play a part in the paper.

Authorship is not rocket science. It is simply about playing fair.

Turning to the last issue of *JHPS*, issue 6.3, I hope you agree that the quality of our papers continues to improve, as I feel we slowly dominate the centre ground of hip preservation research. I was especially intrigued by two very simple papers, yet both have been immensely helpful to my practice. Try the paper by Babazadeh *et al*. [28] on longitudinal versus transverse hip arthroscopy portal cosmesis. Incredibly simple yet incredibly useful. I must clearly change from longitudinal to transverse based on their findings. If you undertake transverse incisions anyway, then you are fine. For me, I must now turn the knife by 90 degrees but otherwise continue as normal. For my other choice, how about the, once again simple, paper from DeFroda *et al*. [29], who used a mini-open incision for their double row gluteus medius repair? Mini-open meant a 4-cm linear incision centred on the tip of the greater trochanter. The authors reported good outcomes at 6 months. Based on these findings, will we all continue with endoscopic repair or is there mileage for mini-open? Naturally, I leave that decision to you.

This issue, number 6.4, is again awash with excellence and I find it difficult to separate one first-class paper from another. I did much enjoy the systematic review of pain management in hip arthroscopy and have been sure my anaesthesiologists have seen it. Thank you to Kolaczko, Knapik and Salata [30] from Ohio for their hard work in bringing this together. They looked at 17 studies, with a total of 1674 patients, and found that nerve blocks were used in 50% and that 68 complications were recorded. There was no significant difference in narcotic consumption, or incidence of complication, based on the modality of pain control used. Again, on the topic of pain relief, another team [31] this time from Chile, South Africa and Australia, looked at the role of compressive cryotherapy in reducing pain after hip arthroscopy. Patients who received compressive cryotherapy reported significantly lower pain scores than those who received standard cryotherapy. There was also a trend towards lower quantities of opioid analgesia being required. Thank you, Klaber, Greef and O’Donnell.

So, as ever, please enjoy this issue of *JHPS*. It is published for you, the hip preservation practitioner, and is filled from cover to cover with brilliance. I commend this issue to you in its entirety.

My very best wishes to you all.
